# The systemic deletion of interleukin-1α reduces myocardial inflammation and attenuates ventricular remodeling in murine myocardial infarction

**DOI:** 10.1038/s41598-023-30662-4

**Published:** 2023-03-10

**Authors:** J. Lugrin, R. Parapanov, G. Milano, S. Cavin, A. Debonneville, T. Krueger, L. Liaudet

**Affiliations:** 1 Service of Adult Intensive Care Medicine, Lausanne University Hospital and University of Lausanne, Lausanne, Switzerland; 2 Service of Thoracic Surgery, Lausanne University Hospital and University of Lausanne, Lausanne, Switzerland; 3 Department Coeur-Vaisseaux, Lausanne University Hospital and University of Lausanne, Lausanne, Switzerland; 4 Laboratoire de Chirurgie Thoracique, Centre des Laboratoires d’Epalinges, Chemin des Boveresses 155, 1066 Epalinges, Switzerland

**Keywords:** Cytokines, Interleukins, Heart failure, Myocardial infarction

## Abstract

Myocardial inflammation following myocardial infarction (MI) is crucial for proper myocardial healing, yet, dysregulated inflammation may promote adverse ventricular remodeling and heart failure. IL-1 signaling contributes to these processes, as shown by dampened inflammation by inhibition of IL-1β or the IL-1 receptor. In contrast, the potential role of IL-1α in these mechanisms has received much less attention. Previously described as a myocardial-derived alarmin, IL-1α may also act as a systemically released inflammatory cytokine. We therefore investigated the effect of IL-1α deficiency on post-MI inflammation and ventricular remodeling in a murine model of permanent coronary occlusion. In the first week post-MI, global IL-1α deficiency (IL-1α KO mice) led to decreased myocardial expression of IL-6, MCP-1, VCAM-1, hypertrophic and pro-fibrotic genes, and reduced infiltration with inflammatory monocytes. These early changes were associated with an attenuation of delayed left ventricle (LV) remodeling and systolic dysfunction after extensive MI. In contrast to systemic *Il1a*-KO, conditional cardiomyocyte deletion of *Il1a* (Cm*Il1a-KO*) did not reduce delayed LV remodeling and systolic dysfunction. In conclusion, systemic *Il1a*-KO, but not *Cml1a-KO*, protects against adverse cardiac remodeling after MI due to permanent coronary occlusion. Hence, anti-IL-1α therapies could be useful to attenuate the detrimental consequences of post-MI myocardial inflammation.

## Introduction

Myocardial infarction (MI) results in extensive death of cardiomyocytes and the subsequent development of a myocardial inflammatory response, which needs to be tightly regulated for appropriate tissue repair^1^. This response is triggered by endogenous molecules, termed damage-associated molecular patterns (DAMPs), released by necrotic cardiac cells to activate innate immune receptors and downstream inflammatory signaling. Several DAMPs likely contribute to these processes, and include HMGB1, fibronectin-EDA, heat shock proteins and nucleic acids, to name a few^2^. In addition, interleukin-1α (IL-1α) has been recently identified as a crucial pro-inflammatory danger signal in the setting of MI^3^. IL-1α is a dual function cytokine (acting as a nuclear transcription regulator and as cell membrane receptor ligand) from the IL-1 family. It is functionally related to IL-1β, and both IL-1α and IL-1β signal through the IL-1R1 receptor complex and trigger pro-inflammatory responses^4^. In contrast to IL-1β, which is primarily induced under pathological conditions, IL-1α is constitutively expressed by numerous cells as a biologically active precursor. As such, IL-1α can function as a pro-inflammatory DAMP upon its passive release by cells undergoing necrotic cell death^5^.

Indeed, we formally identified IL-1α as a DAMP released by necrotic cardiomyocytes, promoting IL-1 receptor-dependent pro-inflammatory signaling in cardiac fibroblasts in vitro^3^, and we found that *Il1a*-KO mice had decreased myocardial and systemic inflammation in an acute model (2 h) of myocardial ischemia/reperfusion (MIR)^3^. These findings echoed previous observations on the pro-inflammatory effects of purified IL-1α in human cardiac myofibroblasts in vitro^6^, and were later substantiated by Mauro et al., who showed that an IL-1α blocking antibody reduced infarct size and preserved left ventricle (LV) function in a mouse model of short term (24 h) MIR^7^. While these previous findings support a role for IL-1α as a myocardial-derived DAMP contributing to the acute post-MI inflammatory response, the role of IL-1α at later stages of cardiac inflammation, repair and remodeling has not been firmly established. These delayed processes are intimately linked to TGF-β signaling, a crucial regulator of cardiac fibroblast activation, myocardial fibrosis and scar formation following MI^8^, and the possible influence of IL-1α on such processes has not been determined. In a study using cardiomyocyte-specific IL-1α KO mouse, Bageghni et al*.* found no difference in post-MI cardiac remodeling after permanent ligation of LAD coronary artery^9^. In contrast, these authors found an improved cardiac function and reduced remodeling in mice with a fibroblast-specific IL-1R1 deletion, suggesting that systemic, but not cardiomyocyte-derived IL-1α, could be detrimental after MI. To address this question, we compared myocardial inflammation and remodeling in wild type (WT) and systemic *Il1a*-knockout (IL-1α KO) mice, and also assessed the influence of a cardiomyocyte-specific deletion of *Il1a* (Cm*Il1a*-KO mice) on delayed cardiac remodeling and dysfunction after MI in a model of permanent ligation of the left anterior descending (LAD) coronary artery.

## Material and methods

### Ethic statement

All the animal experimentations were approved by the Service des Affaires Vétérinaires, Direction Générale de l’Agriculture, de la Viticulture et des Affaires Vétérinaires, Etat de Vaud (Epalinges, Switzerland) under authorization n°VD3327.b. All methods were performed in accordance with the relevant regulations and complied with the ARRIVE guidelines.

### Animals

Male C57BL/6J wild-type mice (Charles River), *Il1a*^−/−^ (obtained from Prof. Yoichiro Iwakura, Tokyo University of Science) and *Il1a*^flox/flox^/*Myh6*-*MerCreMer*^+/−^, were bred under specific pathogen-free conditions before surgery and kept in conventional housing post-surgery. All strains were in C57BL/6J background. The αMHC-MerCreMer mice (Jackson Laboratory, cat. 005657), have the mouse cardiac-specific alpha myosin heavy chain promoter (αMHC, *Myh6*) directing the expression of a tamoxifen-inducible Cre recombinase (MerCreMer) to cardiac myocytes. The *Il1a*-floxed mice (*Il1a*^flox/flox^) were generated by Cyagen (Santa Clara, CA, USA), with LoxP sites spanning exons 3 and 4 of *Il1a* gene (Supplementary Fig. 1). *Il1a*^flox/flox^/*Myh6*-MerCreMer^+/−^ (cardiomyocyte-restricted conditional deletion of *Il1a*) were generated by breeding *Il1a*-floxed with αMHC-MerCreMer mice.

### Tamoxifen induced-deletion of floxed-Il1a in cardiomyocytes

Tamoxifen powder (Sigma) was solubilized in ethanol 100%, diluted at 1:1 ratio with Kolliphor EL (Sigma) and into PBS to obtain a 10 mg/ml stock solution (adapted from^10^), that was diluted (1 mg/ml) in PBS before injection. Conditional *Il1a* deletion in cardiomyocytes was triggered by 4 ip injections of 0.1 ml (0.1 mg) tamoxifen or vehicle over five days (1,1,0,1,1). Since Cre-recombinase expression in cardiomyocytes may cause a transient cardiomyopathy that resolves after 28 days^11^, mice were operated 8 weeks after the last tamoxifen injection. The *Myh6*-*MerCreMer* transgene is known to disrupt the *A1cf* gene^12^. In order to limit the putative side effects of *A1cf* deletion we used *Il1a*^flox/flox^/*Myh6*-MerCreMer^+/−^ (Cre heterozygotes) as commonly done with this model. A timeline of the injection pattern and experimental procedure is shown in Fig. 6b.

### Myocardial Infarction and echocardiography

Myocardial infarction was induced by permanent ligation of the left anterior descending (LAD) coronary artery in anesthetized, intubated and mechanically ventilated male mice (10–12 weeks old), according to our detailed published procedure^13^. Sham animals underwent a similar protocol, except from LAD ligation. After surgery, analgesia was provided by subcutaneous buprenorphine^13^. Different groups of animals were sacrificed by exsanguination under anesthesia (ip ketamine/xylazine, 80/10 mg/kg), after 1, 3, 7, 14, 28 or 42 days. Echocardiography was performed in anesthetized mice (isoflurane 1.5–3%) with a Visualsonics Vevo 2100 apparatus or a Sequoia C256 ultrasound machine with a 15 mHz linear array transducer. Images were obtained before surgery (baseline) and 42 days post-MI in WT and systemic IL-1α KO mice, or 28 days post-MI in tamoxifen (N = 9) or vehicle-treated (N = 9) Il1a^flox/flox^/αMHC-MerCreMer^+/−^ mice. 2D images (B-Mode, parasternal long axis) were acquired to measure LV length and MI length to estimate MI size (MI length/LV length)^14^, as well as to compute LV end-diastolic (LVEDA) and end-systolic area (LVESA) and the fractional area change (FAC), calculated as (LVEDA-LVESA)/LVEDA. The inner LV diameter (LVID) was averaged from 3 measurements in M-mode to compute end-systolic (ESV), end-diastolic (EDV) volumes, LV ejection fraction (LVEF, %), calculated as (EDV-ESV)/EDV × 100, and the fractional shortening (FS, %), calculated as (LVIDd-LVIDs)/LVIDd × 100.

### Histology

Permanent LAD ligation as described above was performed in IL-1α WT (n = 5) and IL-1α KO (n = 6) male C57Bl6/J mice. 14 days after surgery, mice were sacrificed, hearts were embedded in OCT and frozen on dry ice. Hearts were sliced into 5 µm cross sections at 1, 2, 3, 4 and 5 mm below the coronary ligature towards the apex. The slices were stained with picrosirius red to highlight collagen networks and were scanned at 40× magnification with a Hamamatsu NanoZoomer S60 system (Hamatsu Photonics K. K., Shizuoka, Japan). For each section, the LV and collagen surface areas were quantified with ImageJ software (v1.52t). The collagen surface area was expressed as the percentage of LV area in each slice.

### Cell culture conditions and collagen pads contraction assays

Hearts were washed in ice-cold perfusion buffer (in mM: NaCl 130, KCl 5, NaH_2_PO_4_ 0.5, HEPES 10, Glucose 10, butanedione monoxime 10, taurine 10, MgCl_2_ 1), minced with a scalpel and digested into Perfusion Buffer containing 0.2 mg/ml Liberase TL enzyme mix (Roche) and 10 µg/ml DNase I (Roche) at 37 °C with gentle agitation. From the resulting cell suspension, cardiac non-myocyte cells (containing primarily cardiac fibroblasts, CFs) were isolated by differential plating and cultured in DMEM containing 10% FCS and antibiotics, as previously described^3^. For collagen pads experiments, CFs at passage 2 were starved overnight in serum-free DMEM. Type I Collagen pads (Ibidi GMBH, Gräfelfing, Germany) were prepared according to manufacturer’s instructions at a final concentration of 1.5 mg/ml collagen. 0.3 ml of gel mixture containing 2 × 10^5^ cells was distributed in 24 wells plates and incubated 25 min at 37 °C for gelation. Pads were transferred into 6 wells plate, incubated in serum-free DMEM containing or not 10 ng/ml IL-1α, IL-1β and TGF-β for 24 h, and were scanned on a bench-top scanner to assess final size. Areas were measured using ImageJ software (v1.52t). For IL-1α overexpression experiments, HEK 293T cells were cultured under standard conditions (37 °C, 5% CO_2_) in DMEM with 10% FCS and antibiotics. Murine *Il1a* cDNA was cloned into a pCR3-Flag vector using primers described in Supplementary Table 1. Cells were transfected with pCR3-Flag-Il1a using polyethylenimine (PEI, Polysciences Inc.) as a transfectant (1:1.5 DNA/PEI ratio). 24 h after transfection, cells were stimulated with 10 ng/ml TGFβ for 2 h.

### Flow cytometry

Cardiac cells obtained as described above were filtered through a 40 µm mesh, resuspended in FACS buffer (PBS, 2% FCS, 5 mM EDTA), centrifuged and incubated (30 min) with anti-CD16/32 (Fc Block) to block non-specific binding. Cells were stained with antibodies (Supplementary Table 2) for 30 min at 4 °C, and fixed using Foxp3/Transcription Factor Fixation/Permeabilization kit (eBioscence, Carlsbad, USA). After excluding dead cells (based on DAPI coloration) and cell doublets, live cells were first gated according to the expression of CD45/CD11b myeloid markers. CD45^+^/CD11b^+^ cells were then sorted according to expression of the macrophage marker F4/80. F4/80^−^ monocytes were sorted according to Ly6C expression into pro- (Ly6C^hi^) and anti-inflammatory (Ly6C^lo^) monocytes. F4/80^+^ macrophages were sorted according to CD206 into F4/80^+^/CD206^−^ pro-inflammatory (M1) and F4/80^+^/CD206^+^ anti-inflammatory (M2) macrophages. Samples were acquired with a LSR II cytometer (BD Biosciences) using the FACS DIVA software (v8.0) and analysis was done with FlowJo v10 software. Data are presented both as percentage of total live cells and as fold change over sham condition in each genotype. The gating strategy is shown in Supplementary Fig. 1.

### RNA extraction and real-time qPCR

RNA (primary CFs and frozen cardiac tissue) was isolated (TRIzol, Life Technologies, Carlsbad, USA or TriFast, Peqlab, Erlangen, Germany) and reverse transcribed (High Capacity cDNA Reverse Transcription Kit, ThermoFisher Scientific, Vilnius, Lituania). Real-time qPCR was done using the PowerUp SYBR Green Master Mix (ThermoFisher Scientific, Vilnius, Lituania) on 7500 FAST or QuantStudio 12k Flex real-time PCR systems (Applied Biosystems, Foster City, USA). All mRNA expression results were analyzed with the ddCT technique, using *Rps18* gene as a housekeeping gene, and normalized as fold change (FC) over sham WT, unless otherwise specified. All primers used in the PCR experiments are listed in Supplementary Table 1.

### Western Immunoblotting and ELISAs

Cardiac tissue powder, cardiac primary cells and HEK 293T cells were lysed in RIPA buffer containing proteases/phosphatase inhibitors (Roche, Mannheim, Germany), sonicated and centrifuged. Proteins (15–25 µg) were loaded on SDS-PAGE and electro-transferred onto nitrocellulose membranes. Membranes were incubated with primary and secondary antibodies (listed in Supplementary Table 2) and revealed with ECL Reagent (GE Healthcare Life Sciences, Chalfont, United Kingdom). Cardiac tissue levels of IL-6 and MCP-1 were measured using mouse IL-6 DuoSet (DY-406) and mouse CCL2/JE/MCP-1 DuoSet (DY479) ELISA kits (R&D Sytems, Minneapolis, USA). Plasma NT-proBNP was measured with the Mouse NT-proBNP ELISA kit from Elabscience (Houston, USA).

### Statistical analysis

Data are presented as individual values with means ± sem. Comparisons between two groups were made with unpaired two-tailed Student’s *t* test. Analyses of multiple conditions were made with one-way ANOVA followed by Tukey post-test for group comparisons. Two-ways ANOVA with Bonferroni or Dunnet post-hoc tests were used for group comparisons with multiple conditions. For histological analyses, a bilateral paired t test was applied to compare the means of collagen area in each section level. For all statistics, a *p* value < 0.05 was considered significant. We used the PRISM Software (version 8) for all the analyses.

## Results

### IL-1α-KO mice have reduced expression of inflammatory markers in the first week after MI

**Figure 1:**
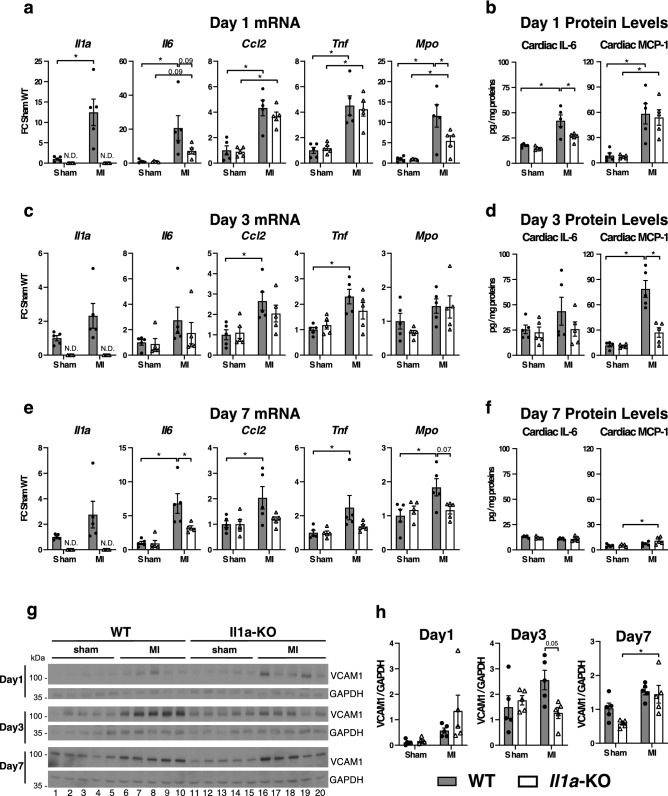
IL-1α-deficiency decreases myocardial inflammation after myocardial infarction. WT and KO mice subjected to chronic LAD ligation (myocardial infarction, MI) or sham surgery were sacrificed after 1, 3 or 7 days and the heart obtained for mRNA, ELISAs and western blot analyses. (**a**) Myocardial mRNA expressions of *Il1a*, *Il6*, *Ccl2/Mcp1*, *Tnf* and *Mpo* pro-inflammatory genes and (**b**) myocardial IL-6 and MCP-1 protein levels at day 1 post sham or MI surgery. (**c**) Myocardial mRNA expressions of *Il1a*, *Il6*, *Ccl2/Mcp1*, *Tnf* and *Mpo* pro-inflammatory genes and (**d**) myocardial IL-6 and MCP-1 protein levels at day 3 post sham and MI surgery. (**e**) Myocardial mRNA expressions of *Il1a*, *Il6*, *Ccl2/Mcp1*, *Tnf* and *Mpo* pro-inflammatory genes and (**f**) myocardial IL-6 and MCP-1 protein levels at day 7 post sham and MI surgery. The mRNA expressions of target genes were detected by RT-PCR, normalized to *Rps18* housekeeping gene expression and expressed as fold change (FC) over sham WT. Cardiac IL-6 and MCP-1 protein expression levels were analyzed by ELISA on tissue lysates and expressed as pg/mg of total proteins. (**g**) Myocardial expression of VCAM-1 detected by western blot 1, 3 and 7 days after sham or MI surgery. GAPDH shown as loading control. (**h**) Quantifications of images shown in **g** expressed as VCAM-1/GAPDH ratios. For all data, MI and sham surgery, N = 5/group. *p < 0.05. N.D., not detected.

At day 1 after MI (Fig. 1a), cardiac mRNA expression of pro-inflammatory *Il1a*, *Il6, Ccl2 (Mcp1), Tnf* and *Mpo* increased in both WT and IL-1α KO mice (except from *Il1a*), the change being less pronounced for *Mpo* and *Il6* (*p* = 0.09) in KO mice. At the protein level, myocardial IL-6 was lower in KO mice, whereas MCP-1 comparably increased in both genotypes (Fig. 1b). At day 3, *Ccl2* and *Tnf* significantly increased only in IL-1α WT but not KO mice (Fig. 1c), and the protein expression of MCP-1 was significantly reduced in IL-1α KO mice (Fig. 1d). At day 7 (Fig. 1c), all genes were overexpressed (mRNA level) only in WT mice, and KO mice displayed reduced *Il6* and *Mpo* (*p* = 0.07) (Fig. 1e). In contrast, no differences were noted with respect to the protein levels of IL-6 and MCP-1 (Fig. 1f). Furthermore, the myocardial expression of VCAM-1, a key adhesion molecule orchestrating leukocyte infiltration in the heart^15^, was significantly lower in IL-1α KO mice at day 3, while it was not different between both genotypes at day 1 and day 7 after MI (Fig. 1g,h). A time-course graph (Supplemental Fig. S3), showing the evolution over time of each mediator, indicates that inflammatory changes peaked at day 1 and progressively decreased thereafter. At day 7, although the mRNA of IL-6 was persistently increased in WT mice, such increase was very modest in comparison to earlier time-points, and was no more accompanied by a parallel increase of IL-6 protein expression.

### IL-1α-deficiency reduces myocardial infiltration with inflammatory monocytes/macrophages at day 3 after MI

**Figure 2:**
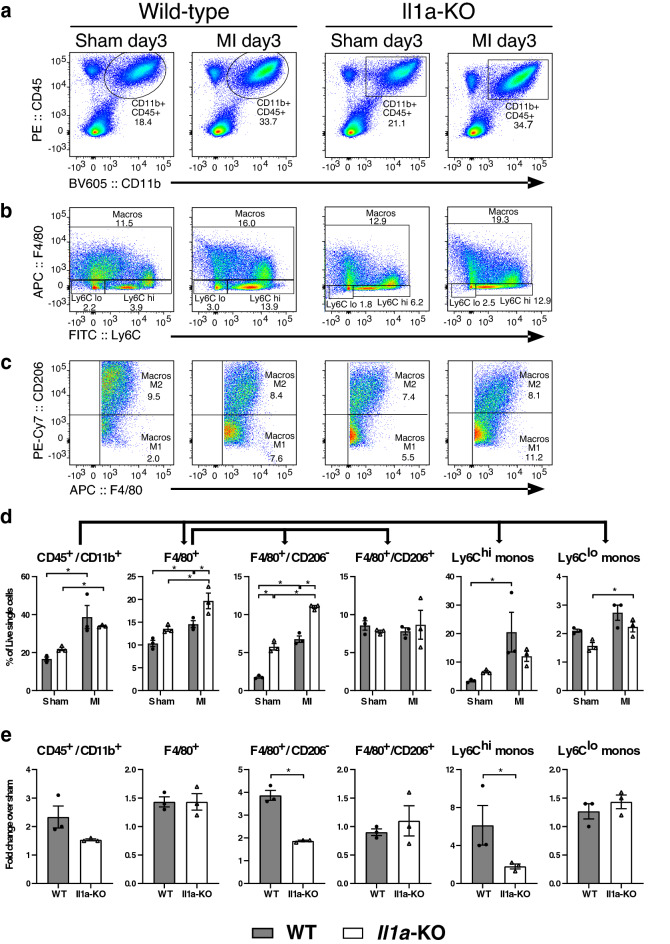
IL-1α-deficiency decreases myocardial infiltration with inflammatory monocytes after MI. Hearts from IL-1α WT and KO mice were obtained 3 days after MI or sham surgery for FACS analysis. Cells expressing the myeloid lineage marker CD45^+^/CD11b^+^ were sorted according to the expression of the macrophage marker F4/80. F4/80^−^ monocytes were sorted according to Ly6C expression into pro- (Ly6C^hi^) and anti-inflammatory (Ly6C^lo^) monocytes. F4/80^+^ macrophages were sorted according to CD206 into F4/80^+^/CD206^−^ pro-inflammatory (M1) and F4/80^+^/CD206^+^ anti-inflammatory (M2) macrophages. (**a**–**c**) Representative FACS analysis (one animal/group) of (**a**) CD45^+^/CD11b^+^ cells, (**b**) F4/80^+^, F4/80^−^/Ly6C^hi^ and F4/80^−^/Ly6C^lo^ cells, (**c**) F4/80^+^/CD206^−^ and F4/80^+^/CD206^+^ cells, expressed as percentages of live cells. (**d**) Bar graphs showing the means ± sem of each cell population (in percentage of live cells) in n = 3 mice/group. (**e**) Owing to differences in cell populations under baseline conditions (sham), data are presented as fold change of each cell population in the myocardium after MI as compared to sham myocardium. N = 3/group. *p < 0.05.

Significant myocardial infiltration with CD45^+^/CD11b^+^ cells occurred at day 3 after MI in both WT and KO mice (Fig. 2a,d). CD45^+^/CD11b^+^ cells were further sorted according to their expression of F4/80 (macrophages) and Ly6C (monocytes), and F4/80^+^ macrophages were then sorted according to their expression of CD206, to distinguish between M1 (F4/80^+^/CD206^−^) and M2 (F4/80^+^/CD206^−^) macrophages. After MI, F4/80^+^ cells (Fig. 2b,d) increased significantly more in KO than WT hearts, an effect related to an increase of F4/80^+^/CD206^-^ cells (Fig. 2c,d) . F4/80^−^/Ly6^hi^ pro-inflammatory monocytes increased only in WT hearts, in contrast to Ly6^lo^ anti-inflammatory monocytes, which increased only in KO hearts (Fig. 2b,d). Owing to baseline differences between WT and KO hearts in the various cell populations (most notably F4/80^+^/CD206^−^ cells, higher in KO hearts), we determined the relative changes of each cell type after MI. As shown in Fig. 2e, IL-1α KO hearts displayed significantly less increase from baseline in both F4/80^+^/CD206^−^ and F4/80^−^/Ly6C^hi^ cells.

### Role of IL-1α on TGFβ signaling in cardiac fibroblasts in vitro and in the infarcted myocardium in vivo

**Figure 3:**
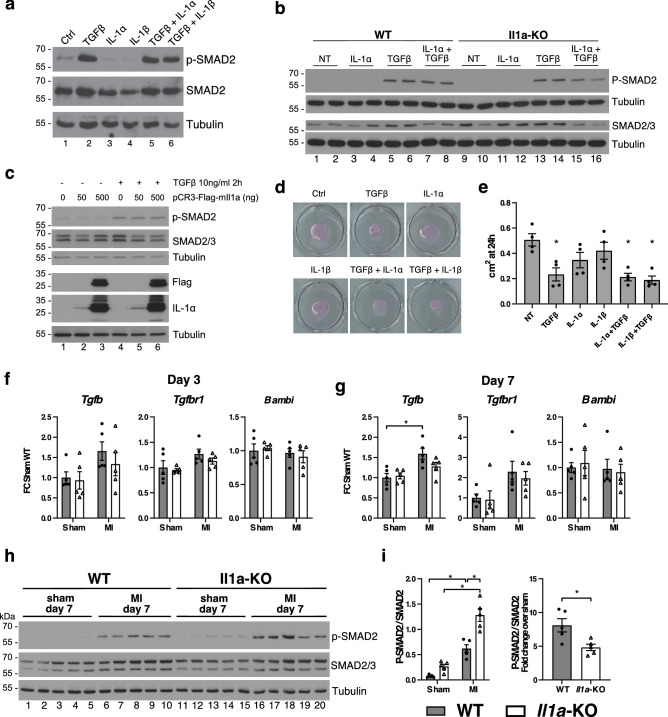
Role of IL-1α on TGFβ signaling in cardiac fibroblasts in vitro and in the infarcted myocardium in vivo. (**a**) Phosphorylation of SMAD2 in adult mouse cardiac fibroblasts cultured for 2 h in the presence of 10 ng/ml TGFβ, 10 ng/ml IL-1α or IL-1β alone or in combination. Blots from one representative experiment (n = 3). (**b**) Protein expression of SMAD2/3 and phospho-SMAD2 in adult mouse cardiac fibroblasts stimulated for 2 h with 10 ng/ml IL-1α or 10 ng/ml TGF-β, alone or in combination. Blots from one representative experiment (n = 3). (**c**) Protein expression of SMAD2/3 and phospho-SMAD2, and expression of IL-1α in HEK 293 T transfected with varying doses of pCR3-Flag-mIl1a vector and stimulated with 10 ng/ml TGF-β for 2 h. Blots from one representative experiment (n = 3). For (**a**–**c**), target proteins and loading controls (tubulin) done on the same blots were grouped together. (**d**) Contraction of collagen pads populated with adult murine cardiac fibroblasts stimulated for 24 h with 10 ng/ml TGFβ, 10 ng/ml IL-1α or IL-1β, alone or in combination. Pads are from one representative experiment. (**e**) Collagen pads surface areas in cm^2^ measured after 24 h stimulation as described in (**d**) (n = 4/condition). (**f**, **g**) mRNA expression of *Tgfb, Tgfbr1* and *Bambi* in the myocardium, three (**f**) and seven (**g**) days after myocardial infarction (MI) or sham surgery (Sham). The mRNA expressions were detected by RT-PCR, normalized to *Rps18* housekeeping genes expression and expressed as fold-change (FC) over sham WT. (**h**) Protein expression and phosphorylation of SMAD2 in the myocardium seven days after MI or sham MI in WT and IL-1α KO mice. Tubulin showed as loading controls. (**i**) Quantification of images shown in (**h**) expressed as p-SMAD2/SMAD2 ratios and as fold change over sham. (**f**–**i**), N = 5/ group. *p < 0.05.

Given the role of TGF-β signaling as a master regulator of CF gene expression and myofibroblast differentiation after MI, we investigated whether exogenous administration, overexpression or deficiency of IL-1α could interfere with this signaling pathway in CFs. TGF-β stimulation of CFs triggered the expected phosphorylation of the SMAD2/3 transcription factors (Fig. 3a). In contrast, IL-1α neither promoted SMAD2 phosphorylation nor influenced the effects of TGF-β in co-stimulation experiments. Similar effects were noted when IL-1α was replaced by IL-1β. Also, stimulation of CFs isolated from IL-1α KO and WT mice with IL-1α and TGF-β, alone or in combination, showed that IL-1α did not promote SMAD2 phosphorylation, nor affected the phosphorylation induced by TGF-β (Fig. 3b). Furthermore, when overexpressed in HEK 293 T cells, IL-1α did not influence SMAD2 phosphorylation, either in the absence or in the presence of TGF-β (Fig. 3c). We then determined the ability of CFs to contract collagen pads following 24 h stimulation with IL-1α, IL-1β and TGF-β, alone or in combination (Fig. 3d,e). While TGF-β significantly promoted pad contraction, there was no significant effects of IL-1α and IL-1β either alone or in combination with TGF-β. We also sought to determine the possible influence of IL-1α on TGF-β signaling in the infarcted myocardium in vivo. We first measured the cardiac mRNA levels of *Tgfb*, *Tgfbr1 (*which encodes for TGF-β receptor type-1) and *Bambi* (BAMBI, BMP and activin membrane-bound inhibitor homolog), a negative regulator of TGF-β signaling. The only noticeable change was a significant increase in the expression of *Tgfb* in the hearts of WT mice at day 7 (Fig. 3f,g). We then assessed the phosphorylation level of SMAD2 at day 7 after MI, and found that it was greater in IL-1α KO and WT mice (Fig. 3h,i). However, since SMAD2 was already slightly phosphorylated under sham conditions in the KO mice, the relative increase after MI was significantly greater in WT than in KO mice.

### Systemic IL-1α deficiency reduces the expression of pro-fibrotic and hypertrophy genes in the first week after MI, and reduces myocardial fibrosis 2 weeks after MI

**Figure 4:**
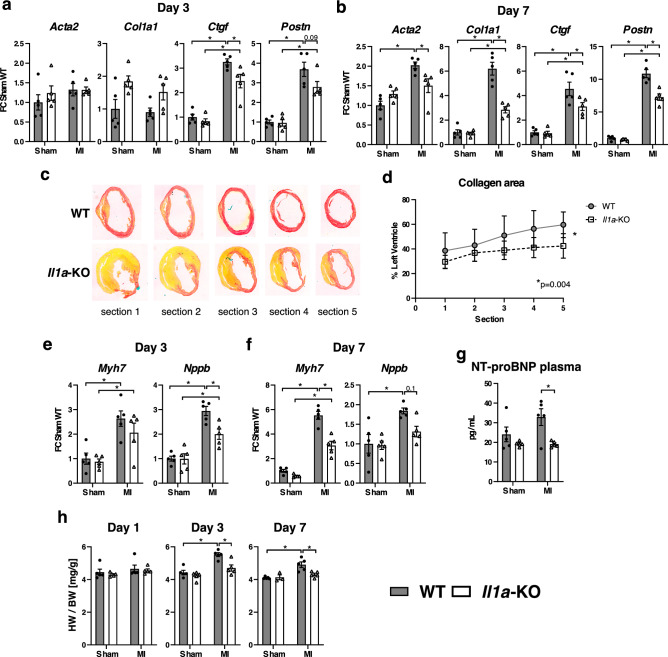
IL-1α-deficiency attenuates pro-fibrotic and hypertrophic signaling in infarcted myocardium. (**a**) mRNA expression of markers of myocardial fibrosis (*Acta2:* alpha-2 smooth muscle actin, *Col1a1*: Collagen α-1 chain, *Ctgf*: *connective tissue growth factor, Postn*: periostin), at day 3 and (**b**) day 7 after MI or sham surgery. (**c**) Representative histological cross-sections of IL-1α WT and IL-1α KO hearts stained for collagen fibers deposits with picrosirius red, 2 weeks after MI. Sections 1–5 were respectively cut 1, 2, 3, 4 and 5 mm below the ligation towards the apex. (**d**) Quantification of collagen surface expressed as percentage of LV surface in IL-1α WT (N = 5) and IL-1α KO hearts (N = 6) for each sections. (**e**, **f**) mRNA expression levels of the hypertrophy markers *Myh7* (Myosin heavy chain β) and *Nppb (*NT-proBNP) at day 3 (**e**) and day 7 (**f**) after MI or sham surgery. mRNA expressions of target genes were detected by RT-PCR, normalized to *Rps18* housekeeping gene expression and expressed as fold-change (FC) over sham WT. (**g**) Plasma levels of NT-proBNP measured by ELISA seven days post-MI or sham surgery. (**h**) Heart weight to body weight ratios 1, 3 and 7 days post-MI or sham surgery. (**a**, **b**, **e**–**h**) N = 5/group. *p < 0.05.

IL-1α promoted early pro-fibrotic changes in the infarcted myocardium, as indicated by the reduced expression of several TGFβ target genes in IL-1α KO mice, both at day 3 (*Ctgf*—connective tissue growth factor, and *Postn*—periostin), and day 7 (*Ctgf*, *Postn*, *Acta2—*alpha-2 smooth muscle actin, and *Col1a1*—Collagen α-1 chain) (Fig. 4a,b). These effects were associated with a significant reduction of collagen fibers deposition in the LV of IL-1α KO mice two weeks after MI, as determined by picrosirius red staining (Fig. 4c,d). In addition, the mRNA expression of two important cardiac hypertrophy genes (*Myh7*, Myosin heavy chain β and *Nppb*, encoding for the prohormone NT-proBNP) increased after MI (Fig. 4e,f), and this change was significantly attenuated in the heart of IL-1α KO mice, at day 3 (*Nppb)* and day 7 (*Myh7*, and, to a lesser extent *Nppb)*. Concomitantly, we noted a significant reduction of circulating NT-proBNP at day 7 post-MI in KO mice (Fig. 4g). The reduced expression of fibrotic and hypertrophic genes in IL-1α KO mice translated in a reduction of the heart weight to body weight ratio (HW/BW) at day 3 and day 7 post-MI (Fig. 4h), consistent with a reduction of early cardiac remodeling in the absence of IL-1α.

### Systemic, but not cardiomyocyte-restricted, deletion of IL-1α attenuates delayed cardiac remodeling after MI

**Figure 5:**
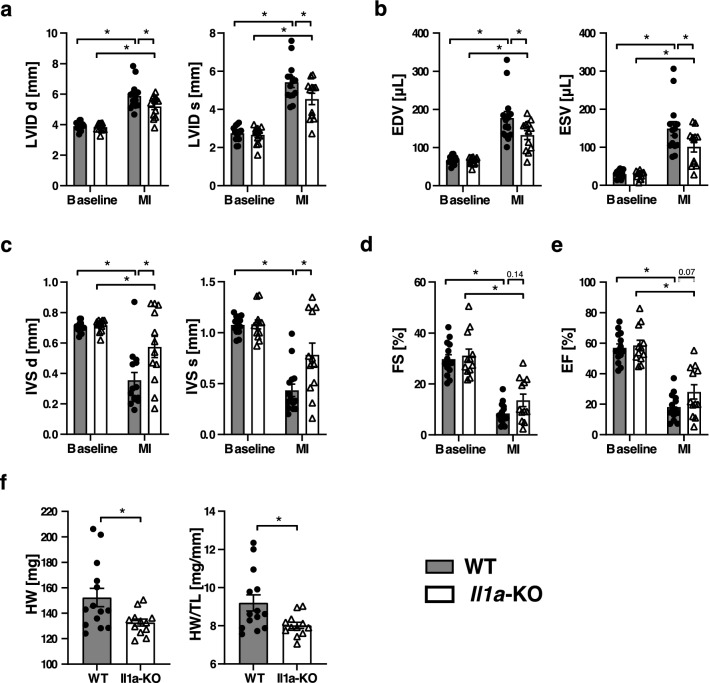
Reduction of left ventricular remodeling and attenuation of systolic dysfunction in IL-1α KO mice six weeks after extensive myocardial infarction. (**a**) LV inner diameter in diastole (LVID d) and systole (LVID s). (**b**) End diastolic (EDV) and end systolic (ESV) LV volumes. (**c**) Thickness of the interventricular septum in diastole (IVS d) and systole (IVS s). (**d**) LV fractional shortening (FS). (**e**) LV ejection fraction (EF). (**f**) Heart weight (HW) and HW to tibial length (HW/TL) ratio. N = 14 WT and 12 IL-1α-KO mice with infarct size > 30% LV. *p < 0.05.

LV remodeling and systolic function was evaluated by echography, performed at baseline and 6 weeks after permanent LAD occlusion. Twenty-one IL-1α WT and 22 IL-1α KO mice underwent surgery. Five animals (2 WT and 3 KO) died before the end of the observation period, and 6 mice without any evidence of MI (1WT and 5 KO) were excluded from the final analysis, which included n = 18 WT and n = 14 KO mice. Two sets of analyses were done, the first one including all the animals, the second one including only animals with an infarct size > 30%, evaluated by echocardiography (n = 14 WT and n = 12 KO). Indeed, it is currently recommended that MI size < 30% should be excluded because of the absence of significant LV remodeling^16^. The results of these two analyses are shown in Table 1 (two groups comparison using t test), and data in mice with infarct size > 30% are also depicted in Fig. 5 (Two Way ANOVA). The different echographic variables, as well as heart weight (HW) and the ratio of HW to tibia length (HW/TL), were not significantly different between WT and KO mice when comparing data from all mice. In contrast, in the presence of large infarcts, KO mice displayed significant reduction of LV remodeling, as indicated by less LV dilation (Fig. 5a,b) and interventricular septum thinning (Fig. 5c). Furthermore, KO mice had a tendency for better preservation indices of LV systolic function, including fractional shortening (Fig. 5d , *p* = 0.14 ANOVA, *p* = 0.05, *t* test) and ejection fraction (Fig. 5e , *p* = 0.07 ANOVA, *p* = 0.06, t test). The reduction of LV remodeling was further substantiated by the smaller heart weight and HW/TL ratio in KO mice (Fig. 5f). The same differences persisted (except from ejection fraction and fractional shortening) when only animals with infarct size > 40% LV were compared (Table 1).

**Table 1:** Hemodynamic data and heart weight in WT and IL-1α KO mice 6 weeks after MI.

	All Infarcts (n = 32)			Infarct size > 30% (n = 26)			Infarct size > 40% (n = 17)		
	WT (n = 18)	KO (n = 14)	*p*	WT (n = 14)	KO (n = 12)	*p*	WT (n = 9)	KO (n = 8)	*p*
Infarct size, % LV	42 ± 2	39 ± 3	0.50	45 ± 2	42 ± 2	0.24	50 ± 2	46 ± 2	0.14
HR, bpm	533 ± 15	540 ± 17	0.75	522 ± 17	545 ± 18	0.36	515 ± 22	535 ± 23	0.59
CO, ml/min	15.1 ± 1.5	17.3 ± 1.7	0.35	13.7 ± 1.6	16.8 ± 1.8	0.19	12.2 ± 2.0	14.8 ± 2.1	0.38
SV, μl	29.5 ± 2.6	32.7 ± 2.9	0.42	27.2 ± 2.8	31.8 ± 3.0	0.26	24.9 ± 3.4	28.3 ± 3.6	0.51
LVPWd, mm	0.63 ± 0.06	0.61 ± 0.07	0.85	0.60 ± 0.07	0.58 ± 0.08	0.86	0.51 ± 0.08	0.51 ± 0.08	0.98
LVPWs, mm	0.84 ± 0.08	0.78 ± 0.09	0.57	0.78 ± 0.09	0.73 ± 0.10	0.69	0.65 ± 0.11	0.65 ± 0.12	0.98
IVSd, mm	0.45 ± 0.06	0.61 ± 0.07	0.07	0.36 ± 0.03	0.57 ± 0.06	**0.01**	0.36 ± 0.08	0.51 ± 0.08	0.20
IVSs, mm	0.58 ± 0.09	0.84 ± 0.10	0.06	0.43 ± 0.09	0.78 ± 0.09	**0.01**	0.43 ± 0.11	0.67 ± 0.11	0.14
LVIDd, mm	5.53 ± 0.22	5.03 ± 0.25	0.14	5.89 ± 0.22	5.19 ± 0.23	**0.04**	6.32 ± 0.23	5.46 ± 0.25	**0.02**
LVIDs, mm	4.95 ± 0.28	4.32 ± 0.32	0.16	5.42 ± 0.27	4.54 ± 0.29	**0.03**	5.92 ± 0.30	4.93 ± 0.31	**0.03**
LV EDV, μl	157 ± 14	124 ± 16	0.13	177 ± 15	133 ± 16	**0.04**	206 ± 17	147 ± 18	**0.03**
LV ESV, μl	126 ± 15	92 ± 17	0.15	149 ± 16	101 ± 17	**0.05**	180 ± 19	119 ± 20	**0.04**
LVEDA, mm^2^	36.9 ± 1.9	32.9 ± 2.1	0.17	40.0 ± 1.9	34.1 ± 2.0	**0.04**	43.4 ± 2.1	63.5 ± 2.2	**0.04**
LVESA, mm^2^	32.8 ± 2.1	27.7 ± 2.4	0.13	36.2 ± 2.1	29.2 ± 2.3	**0.03**	40.2 ± 2.3	32.2 ± 2.5	**0.03**
LV length, mm	22.9 ± 0.5	21.8 ± 0.6	0.17	23.7 ± 0.5	22.1 ± 0.6	**0.04**	24.6 ± 0.6	22.9 ± 0.60	**0.04**
FAC, %	13 ± 2	17 ± 2	0.09	10 ± 2	15 ± 2	**0.03**	8 ± 1	12 ± 1	**0.04**
FS, %	11.7 ± 1.9	15.3 ± 2.2	0.24	8.4 ± 1.7	13.6 ± 1.9	**0.05**	6.4 ± 1.7	10.2 ± 1.9	0.16
EF, %	24 ± 4	31 ± 4	0.24	18 ± 3	28 ± 4	0.06	14 ± 4	22 ± 4	0.16
Heart weight, mg	145 ± 5	132 ± 6	0.09	152 ± 6	133 ± 6	**0.02**	162 ± 7	133 ± 8	**0.01**
HW/TL	8.8 ± 0.3	8.0 ± 0.3	0.10	9.2 ± 0.3	8.0 ± 0.4	**0.02**	9.8 ± 0.4	8.0 ± 0.5	**0.01**

Data from all animals and data form animals with infarct size > 30% and > 40% LV. P values calculated by *t* test (two group comparisons). Values in bold indicate significant differences between WT and KO mice.

*BPM* beats per minute, *CO* cardiac output, *FAC* fractional area change, *FS* fractional shortening, *EF* ejection fraction, *HR* heart rate, *HW/TL* heart weight/tibia length, *IVSd* interventricular septum thickness, diastole, *IVSs* interventricular septum thickness, systole, *LV* left ventricle, *LVEDA* LV end diastolic area, *LVESA* LV end systolic area, *LV EDV* LV end diastolic volume, *LV ESV* LV end systolic volume, *LVIDd* LV inner diameter, diastole, *LVIDs* LV inner diameter, systole, *LVPWd* LV posterior wall thickness, diastole, *LVPWs* LV posterior wall thickness, systole, *SV* stroke volume.

**Figure 6:**
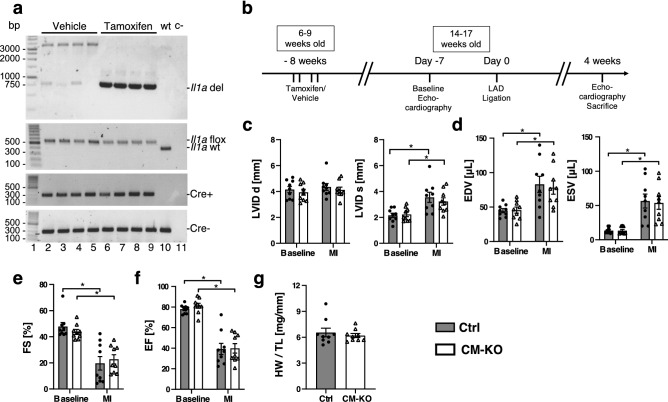
Specific IL-1α-deficiency in cardiomyocytes does not influence left ventricle remodeling four weeks after myocardial infarction. (**a**) Specific *Il1a* deletion in cardiomyocytes of *Il1a*^fl/fl^/*Myh6*-*Cre*^+/−^ mice treated with tamoxifen. (**b**) Timeline of tamoxifen-induced deletion, coronary ligation and echographic assessments. (**c**) Left ventricle inner diameter in diastole (LVID d) and systole (LVID s). (**d**) End diastolic (EDV) and end systolic (ESV) left ventricular volumes. (**e**) Left ventricular fractional shortening and (FS) and (**f**) ejection fraction (EF) at baseline and four weeks after MI. (**g**) Heart weight (HW) over tibia length (TL) ratio. N = 9 mice/group.

We then sought to determine whether comparable effects on cardiac remodeling would occur in mice with a conditional deletion of IL-1α in cardiomyocytes. The conditional deletion was verified by PCR for *Il1a* floxed allele in cardiac tissue obtained from vehicle or tamoxifen-treated αMHC-MerCreMer^+/−^/Il1a^flox/flox^ mice (Fig. 6a). The experimental protocol for tamoxifen or vehicle injections, MI surgery and echocardiographic studies is depicted in Fig. 6b. In these experiments, echographic determination of MI size was not performed. Thus, we included all the animals in one single analysis. Increased LV diameter (Fig. 6c) and volume (Fig. 6d) comparably developed 28 days after MI in vehicle or tamoxifen-treated mice. Also, the depression of LVFS and LVEF (Fig. 6e,f), as well as the HW/TL ratio (Fig. 6g), were strictly comparable without any significant differences between the two groups of mice.

## Discussion

The main results of our study indicate that the systemic deficiency of IL-1α reduces early myocardial inflammation and expression of pro-fibrotic and hypertrophic genes, resulting in long-term improvement of LV remodeling after MI induced by permanent coronary artery ligation. In contrast, cardiomyocyte-restricted deletion of IL-1α does not prevent LV remodeling and systolic dysfunction after MI.

Global IL-1α deficiency was accompanied by a modulation of the acute inflammatory response following MI, as indicated by reduced expression of the *il6* and *Mpo* genes, as well as reduced protein expression of IL-6 and MCP-1 in the myocardium. These effects were essentially observed at day 1 (mRNA levels) and day 1–3 (protein levels), which supports a key role of IL-1α in the hyperacute innate immune response triggered by myocardial necrosis^17^. It is particularly noticeable that IL-6 has been associated with increased morbidity and mortality after MI^18^, and that blocking IL-6 signaling with tocilizumab reduces systemic inflammation and myocardial injury at the acute phase of MI^18,19^. Reduced IL-6 expression in IL-1α KO mice therefore indicates that IL-1α is instrumental in triggering this major pathway of myocardial inflammation and damage after MI.

Besides IL-6, the reduction of myocardial MCP-1 in IL-1α KO mice was another important finding, owing to the known prognostic role of this major monocyte-attracting chemokine in the acute and chronic phases after acute coronary syndrome^20,21^. The recruitment of monocytes to the myocardium under various stress conditions is dependent on their expression of CCR2, the MCP-1 receptor^22^, and it has been reported that adverse cardiac remodeling after MI is reduced in mice deficient in MCP-1^23^ or following CCR2 silencing^24^. In addition to MCP-1, the adhesion molecule VCAM-1 is another mediator essential to govern leukocyte infiltration in the inflamed heart^15^, and we found that it was also significantly, albeit transiently (at day 3 only), downregulated after MI in IL-1α deficient mice. Reduced MCP-1 and VCAM-1 expression in the absence of IL-1α was accompanied by a decreased myocardial infiltration by pro-inflammatory monocytes (Ly6C^hi^) at day 3 post-MI in IL-1α KO mice. Although these inflammatory mononuclear cells play an important role to eliminate necrotic debris within the infarcted myocardium, their excessive activation has been associated with averse cardiac remodeling, through the release of cytokines and proteolytic enzymes, amplifying the initial inflammatory response, degrading the extracellular matrix and promoting fibrosis^25,26^. Collectively, these findings support a key mechanistic role of IL-1α in the recruitment of inflammatory cells within the infarcted myocardium. This assumption is in agreement with a recent report by Schunk et al., who reported that IL-1α enhances the expression of VCAM-1 and leukocyte-endothelial adhesion, increases homing of monocytes at sites of vascular injury, and promotes the accumulation of macrophages and neutrophils in inflamed tissue in vivo^27^.

The acute inflammatory response after MI is followed by a proliferative phase characterized by the activation of myofibroblasts and the progressive formation of a fibrotic scar, under the control of TGF-β, which signals through receptor-activated SMAD proteins, primarily SMAD2 and SMAD3^28^. These processes must be tightly regulated to ensure proper myocardial healing, and may become maladaptive when the response is either insufficient (risk of cardiac rupture) or excessive (promotion of adverse ventricular remodeling)^29^. In vitro, we found that both IL-1α and IL-1β did not influence SMAD2 phosphorylation in CFs, and that SMAD2 phosphorylation in response to TGF-β was comparable in WT and IL-1α KO CFs. Also, IL-1α overexpression (in HEK 293 T cells) did not trigger SMAD phosphorylation, and neither IL-1α nor IL-1β exerted significant effects on the contraction of collagen pads. Collectively, these observations indicate that the activation of the IL-1 receptor does not directly interfere with TGFβ signaling and does not promote a contractile phenotype in CFs in vitro, in agreement with data indicating that both IL-1α^30^ and IL-1β^31^ do not induce contraction of CFs in vitro.

In vivo, at day 7 after MI, the absence of IL-1α did not influence the expression of TGFβ, the TGFβ receptor and the TGFβ decoy receptor BAMBI, a negative regulator of TGFβ signaling^31^. This is at variance with IL-1β, which has been shown to increase the expression of BAMBI and reduce the expression of the TGFβ co-receptor endoglin in CFs^31^, pointing to distinct actions of both IL-1 isoforms during the proliferative phase of healing after MI. With respect to SMAD2/3, we found that its phosphorylation status was greater in IL-1α KO mice after MI. However, the relative increase of SMAD phosphorylation after MI was significantly lower than in WT mice, owing to the presence of a slightly higher baseline (sham) level of phosphorylated SMAD in IL-1α KO mice. Therefore, while IL-1α does not have direct effects on TGFβ signaling in vitro, it may contribute to promote SMAD phosphorylation after MI in vivo, through mechanisms which remain to be elucidated.

Furthermore, IL-1α KO mice displayed a significant reduction of genes involved in pro-fibrotic response after MI (Day 3 and especially Day 7). These genes included *Acta2*, which encodes for α-smooth muscle actin, the prototypical marker of differentiated myofibroblasts^32^, *Ctgf* (Connective tissue Growth Factor), a surrogate marker for activated fibroblasts^33^, as well as *Col1a1* (type 1 collagen) and *Postn* (periostin), two important structural proteins from the extracellular matrix^34^. Accordingly, IL-1α KO mice displayed significantly less collagen fibers deposition in the LV at 2 weeks after MI, implying that IL-1α promotes early pro-fibrotic changes in the healing myocardium. In addition to the aforementioned genes, IL-1α KO mice also had reduced expression of the hypertrophy gene *Myh7* (β-myosin heavy chain)^35^, a finding consistent with the cardiac hypertrophy reported in transgenic mice overexpressing IL-1α^36^, as well as *Nppb,* which encodes for the B-type natriuretic peptide. The latter change was reflected by a marked decrease of the plasma levels of its N-terminal pro-hormone NT-proBNP, a surrogate biomarker of cardiac volume and pressure with prognostic implication during the progression of heart failure^37^. Altogether, these data indicate that IL-1α represents an important signal for early post-MI fibrotic and hypertrophic ventricular remodeling, leading to enhanced myocardial wall stress, an assumption that was further confirmed by the suppressed increase of heart weight at day 3 and day 7 after MI in IL-1α KO mice.

On a functional standpoint, the reduction of early myocardial remodeling afforded by systemic IL-1α deficiency translated into a delayed amelioration of LV structure and function, as evidenced by less LV dilation and myocardial thinning, as well as slightly better preserved systolic function. These effects of IL-1 deficiency were observed in mice with large infarct size, known to be associated with significant LV remodeling^16^. These findings concur with a previous study by Abbate et al. in a similar model of permanent LAD occlusion, reporting that systemic deletion of the IL-1 receptor reduced LV remodeling and dysfunction seven days post-MI^38^. Also, the same investigators reported that Anakinra, an antagonist of the IL-1 receptor, favorably influenced LV remodeling 14 days after MI in human patients^39^. According to a recent study by Bageghni et al., these detrimental actions of IL-1R-mediated signaling implicate primarily the cardiac fibroblast (CF), as shown by the significant reduction of adverse cardiac remodeling after MI in mice with a conditional deletion of IL-1R1 in CFs^9^. Taken together, these data underscore the critical importance of IL-1/IL-1R signaling in promoting post-MI LV remodeling and dysfunction. In this respect, the role of inflammasome-dependent IL-1β generation has been put at the foreground^40^, and the possibility of a specific role of IL-1α has been generally disregarded. Our findings, however, do support a previously unappreciated role of this IL-1 isoform in post-MI cardiac remodeling and fibrosis, suggesting that specific IL-1α antagonists could represent a possible therapeutic approach to limit adverse post-MI remodeling. Such an approach would have the advantage to avoid the risk of immune suppression, which may be associated with IL-1β or IL-1R1 antagonism^41,42^.

Interestingly, at variance with our results, Razin et al. recently reported that global IL-1α deletion resulted in enhanced LV dilation and systolic dysfunction 3 and 24 days after MI, by preventing IL-1α-dependent protection against CF apoptosis^43^. It must be underscored that these data were obtained, in part, using female mice, suggesting possible gender-related differences in the effects of IL-1α after MI. Also, cardiac dilation and dysfunction already occurred at a very early time-point (3 days) after MI, supporting massive MI rather than adverse delayed remodeling as the predominant mechanism. Finally, LV dilation and ventricular wall thinning did not occur in IL-1α competent mice, and functional studies were not done beyond 24 days. Therefore, comparisons with our study are difficult, given that we assessed LV morphology and function after 42 days, and that we observed marked LV dilation and thinning in IL-1α WT animals.

We previously reported that IL-1α is released as a DAMP from necrotic cardiomyocytes and triggers inflammatory responses in an acute model of myocardial ischemia and reperfusion^3^. In a recent study, Bageghni and colleagues determined whether suppression of IL-1α in cardiomyocytes could improve the adverse cardiac remodeling and function after permanent MI. They found that cardiomyocyte-specific deletion of *Il1a* (Cm-KO) did not confer any amelioration, in contrast to their findings in cardiac fibroblasts-specific IL-1R1-deficient mice^9^, as discussed previously. By using a similar model of *Il1a* conditional deletion in cardiomyocytes, we did not observe any significant difference regarding cardiac remodeling and function between Cm-KO and WT mice 4 weeks after MI, confirming the results of Bageghni et al. Collectively, these findings suggest a dual function of IL-1α during MI. At the hyper-acute phase, IL-1α primarily acts as a DAMP to initiate early inflammatory changes in response to myocardial necrosis. Additional studies evaluating very early myocardial inflammation in Cm-KO mice would here be important to further support this hypothesis, and such experiments should be performed both in permanent or transient (ischemia–reperfusion) MI models. Subsequently, IL-1α functions as a cytokine from non-cardiomyocyte origin contributing to the process of adverse myocardial remodeling leading to chronic LV dysfunction.

Circulating monocytes and bone-marrow derived macrophages may be here a major source of systemic IL-1α. In support of this assumption, Fettelschoss et al*.* showed that murine monocytes, bone-marrow derived macrophages and dendritic cells express cell surface-associated IL-1α and secrete mature IL-1α in vitro and in vivo^44^. Also, Freigang et al*.* showed that macrophages from myeloid origin released IL-1α to promote vascular inflammation in a murine model of atherosclerosis^45^. Finally, Schunk et al. recently reported that IL-1α expressed at the surface of circulating monocytes in patients experiencing acute MI was associated with later atherosclerotic cardiovascular events, possibly via VCAM-1-dependent endothelial leukocyte adhesion^27^. Future studies using single-cell RNA sequencing in the infarcted myocardium as well as experiments in bone marrow chimeric mice would be useful to precise the cellular sources of IL-1α in the conditions of our study.

We acknowledge several limitations to our study. First, we used a permanent model of LAD occlusion and did not address the role of IL-1α in a reperfused model. Although current recommendations indicate that permanent coronary occlusion should be the model of choice to study LV remodeling and progression to heart failure^16^, we cannot rule out that distinct outcomes would have developed in a model of reperfused MI. Second, we did not evaluate inflammation and fibrotic signaling in conditional KO mice. However, in the absence of any influence on LV size and function 4 weeks after MI, a significant influence of conditional IL-1α deletion on these signals appears highly unlikely.

In conclusion, the systemic deletion of IL-1α is associated with a downregulation of the acute inflammatory changes after MI induced by permanent coronary occlusion. These anti-inflammatory effects are followed by a decreased expression of pro-fibrotic genes and by a reduction of myocardial hypertrophic responses, resulting in the attenuation of delayed adverse ventricular remodeling and systolic dysfunction. In contrast, the specific deletion of IL-1α in cardiomyocytes does not confer any protection against myocardial remodeling and systolic dysfunction. These results support a mechanistic role of IL-1α as a systemically released cytokine, but not as a cardiomyocyte-released DAMP, in the process of ventricular remodeling leading to heart failure after myocardial infarction.

## Supplementary Information

Supplementary Information 1.

Supplementary Information 2.

## Data Availability

The datasets used in this study are available from corresponding author upon reasonable request.
