# Supplementary material for: The systemic deletion of interleukin-1α reduces myocardial inflammation and attenuates ventricular remodeling in murine myocardial infarction
Source: Sci Rep. 2023 Mar 10;13:4006. doi: 10.1038/s41598-023-30662-4 (PMC10006084; doi:10.1038/s41598-023-30662-4)
Supplement: Supplementary file 1 — Supplementary Information 1. [file 41598_2023_30662_MOESM1_ESM.pdf]

The systemic deletion of interleukin-1α reduces myocardial inflammation and attenuates ventricular remodeling in murine myocardial infarction

Short title: IL-1α deficiency reduces post-MI inflammation and remodeling

Lugrin J.[1,2], Parapanov R.[1,2], Milano G. [3], Cavin S.[2], Debonneville A. [2], Krueger T. [2], Liaudet L.[1]

[1] Service of Adult Intensive Care Medicine, [2] Service of Thoracic Surgery, [3] Department Coeur-Vaisseaux, Lausanne University Hospital and University of Lausanne, Lausanne, Switzerland

Supplementary Figure 1. Generation of mice with *Il1a*-floxed allele spanning exons 3 and 4 of *Il1a* gene. *Il1a*-floxed mice were generated by Cyagen Biosciences Inc. (Santa Clara, CA, USA) using the following targeting strategy. LoxP sites spanning exons 3 and 4 was done by homologous recombination with a targeting vector. Neomycin resistance cassette (Neo^r) was removed by Flp recombination producing the $Il1a^{fl}$ allele. Upon Cre recombinase expression and Cre recombination, exons 3 and 4 are deleted producing the KO allele.

Supplementary Figure 2. Gating strategy for FACS analysis. Cell debris, cell doublets and dead cells (according to DAPI staining) were excluded from analysis. Analysis was done on single live cells. Monocytes/macrophages were identified as $CD11b^+$/$CD45^+$ positive cells. Macrophages were characterized by expression of F4/80 and monocytes as F4/80^- population. Pro-inflammatory monocytes were $Ly6C^+$ and anti-inflammatory monocytes $Ly6C^-$. Macrophages were further characterized as M1 pro-inflammatory ($CD206^-$) and M2 anti-inflammatory ($CD206^+$) macrophages.

Supplementary Figure 3. Time course expression of pro-inflammatory genes mRNA and pro-inflammatory cytokines during the first week after MI. Myocardial mRNA expressions of (**a**) *Il1a*, (**b**) *Il6*, (**c**) *Ccl2/Mcp1*, (**d**) *Tnf* and (**e**) *Mpo* pro-inflammatory genes at days 1, 3 and 7 post sham and MI surgery. mRNA expressions of target genes were detected by RT-PCR, normalized to *Rps18* housekeeping gene expression. (**f**) Myocardial IL-6 and (**g**) MCP-1 proteins levels at days 1, 3 and 7 post sham and MI surgery. Proteins from tissue lysates were analyzed by ELISA and expressed as pg/mg of total proteins. N=5/group, * $p < 0.05$. N.D., not detected.

SUPPLEMENTARY FIGURE 1

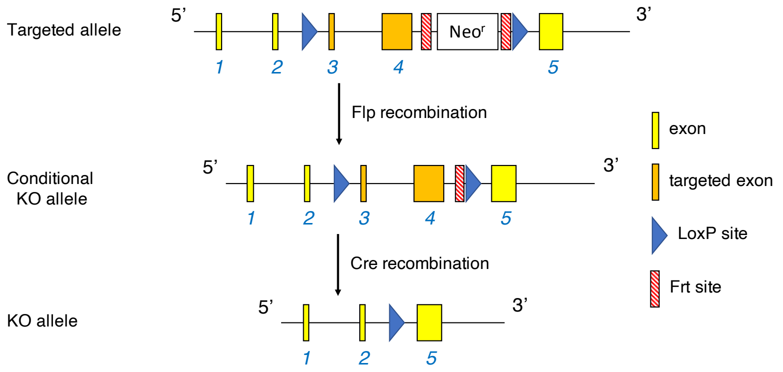

Supplementary Figure 2

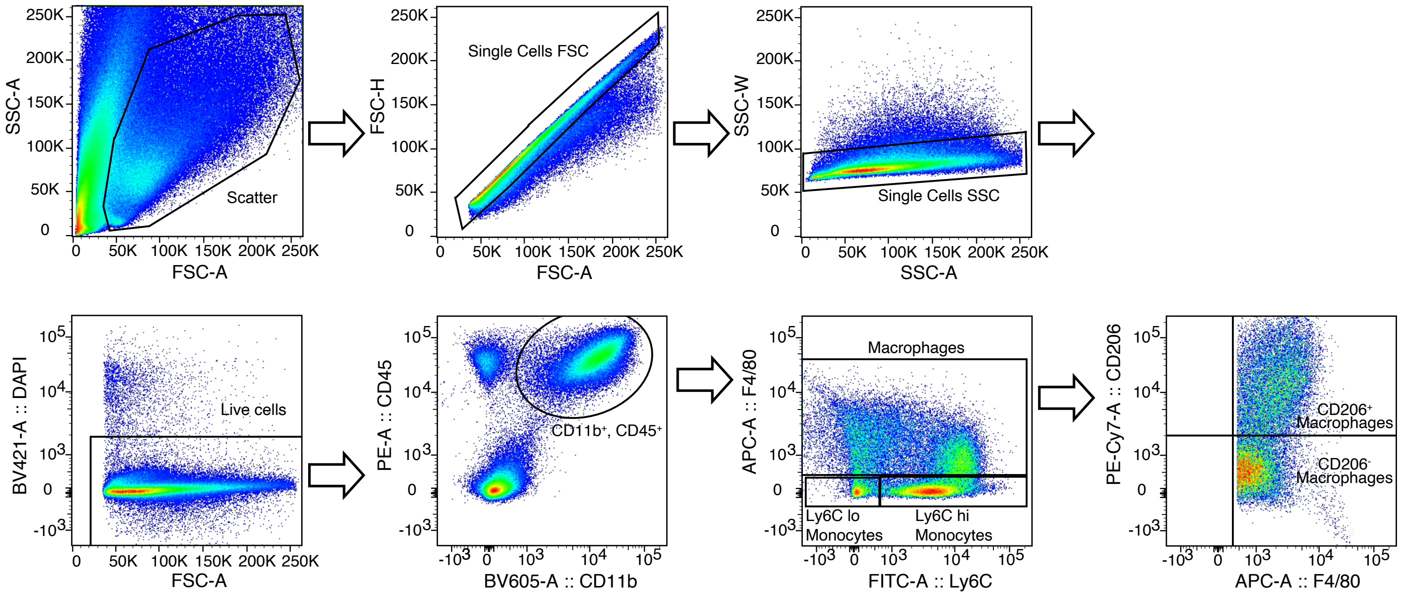

Supplementary Figure 3

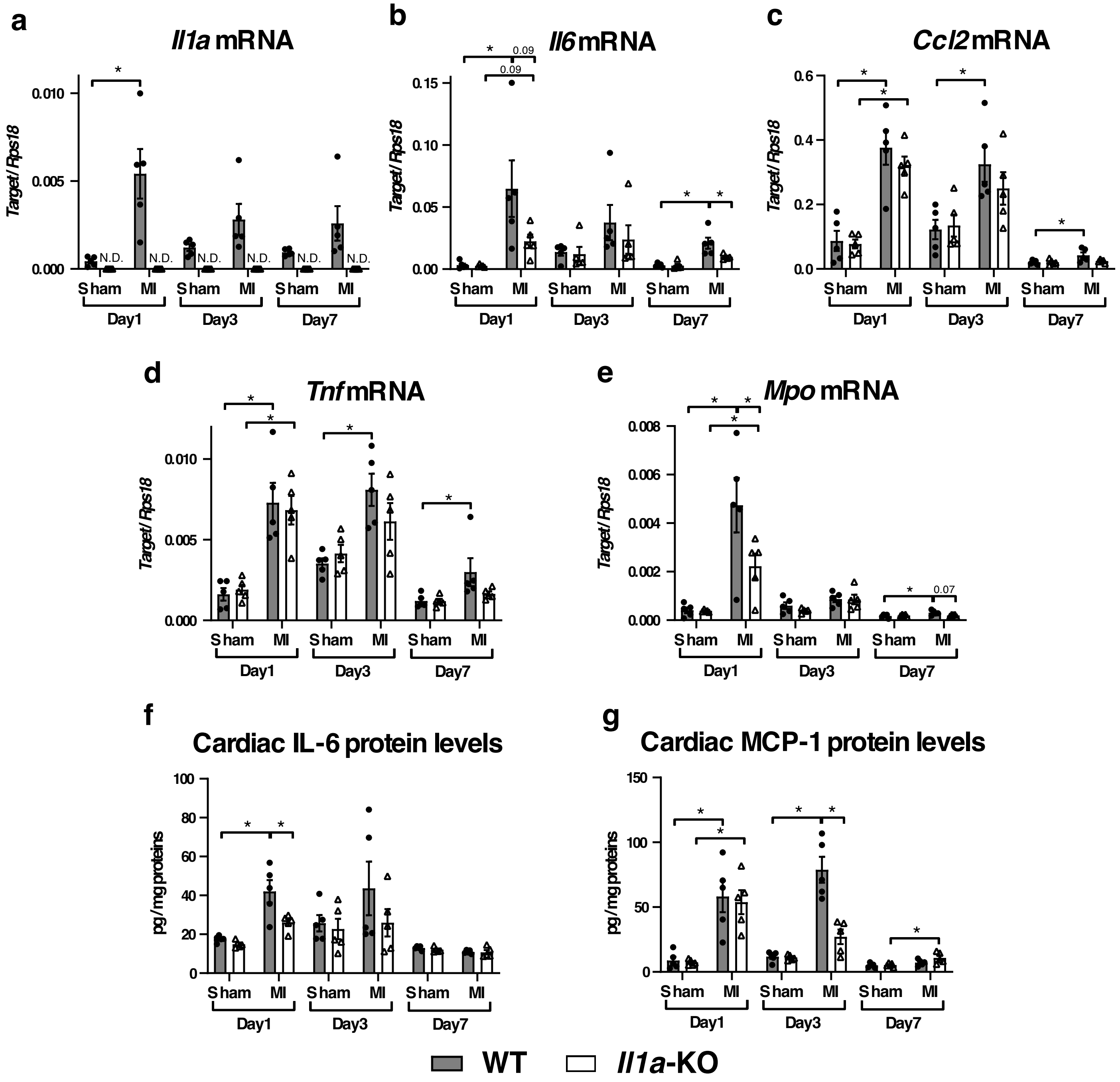

Supplementary Table 1 : Oligonucleotides

Gene name	Protein	Forward sequence (5'-3')	Reverse Sequence (5'-3')	Use
Acta2	α-SMA	TTCATTGGGATGGAGTCAGCG	AATGCCTGGGTACATGGTGG	RT-PCR
Bambi	BMP and activin membrane-bound inhibitor homolog	ACAGCTTTCACGGACACCAT	TTGCCGCATTTTGTCACAGG	RT-PCR
Ccl2	CCL2/MCP-1	GCACCAGCACCAGCCAACTCT	GCAGGCCCAGAAGCATGACAGG	RT-PCR
Col1a1	Collagen alpha-1(I) chain	GAGAGGTGAACAAGGTCCCG	AAACCTCTCTCGCCTCTTGC	RT-PCR
Ccn2/Ctgf	CCN family member 2/Connective tissue growth factor	CACTCTGCCAGTGGAGTTCA	AGATGTCATTGTCCCCAGGA	RT-PCR
Il1a	Interleukin-1α	GCAACGGGAAGATTCTGAAG	TGACAAACTTCTGCCTGACG	RT-PCR
Il6	Interleukin-6	GTGGCTAAGGACCAAGACCA	ACCACAGTGAGGAATGTCCA	RT-PCR
Mpo	Myeloperoxidase	CACCCTCTTTGTTCGAGAGC	AGAGCTTCTCCCCATTCCAT	RT-PCR
Myh7	Myosin-7/Myosin heavy chain 7	GGCCCTTTGACCTCAAGAAAGA	TCACCGTCTTGCCATTCTCC	RT-PCR
Nppb	Natriuertic peptides B / Brain natriuretic peptide B	CTGAAGGTGCTGTCCCAGAT	CCTTGGTCCTTCAAGAGCTG	RT-PCR
Postn	Periostin	GCAAACCACTTTCACCGACC	CGTTGGTCCATGCTCAGAGT	RT-PCR
Rps18	40S ribosomal protein S18	ACTTTTGGGGCCTTCGTGTC	GCCCAGAGACTCATTTCTTCTTG	RT-PCR
Tgfb1	Transforming growth factor beta-1 proprotein	CACCGGAGAGCCCTGGATA	TGTACAGCTGCCGCACACA	RT-PCR
Tgfbr1	TGF-beta receptor type-1	AGCGTTCATGGTTCCGAGAG	TCTGACACCAACCACAGCTG	RT-PCR
Tnf	Tumor necrosis factor	TGTTGCCTCCTCTTTTGCTT	TGGTCACCAAATCAGCGTTA	RT-PCR

Gene name	Target	Forward sequence (5'-3')	Reverse Sequence (5'-3')	Use
Myh6-Cre	Wild-type allele	TCTATTGCACACAGCAATCCA	CCAACTCTTGTGAGAGGAGCA	genotyping
Myh6-Cre	Mutant allele (Cre expressing)	TCTATTGCACACAGCAATCCA	CCAGCATTGTGAGAACAAGG	genotyping
Il1a-floxed	Region spanning LoxP site located in 3' of exon 4	TAACTATAAGGGAAAGGTCAGTAGG	GTTTTGGAGCTTAGAGAGAGTGA	genotyping
Il1a-del	Amplification of a 750bp fragment upon deletion of exons 3 and 4	ACAGCCACTGGTGTTCTCTT	GTTTTGGAGCTTAGAGAGAGTGA	genotyping

Gene name	Target	Sequence (5'-3')	Comment	Use
Il1a	Murine *Il1a*	AAAgaattcATGGCCAAAGTTCCTGACTTGT	BamHI restriction site	cloning
Il1a	Murine *Il1a*	AAAgcggccgcTTATGATATCTGGAAGTCTGTCATAGAGG	NotI restriction site	cloning

Supplementary Table 2 : Antibodies used for western blotting and flow cytometry.

Western Blot Antibodies	Brand	Catalog n°	Dilution
α-Tubulin	Adipogen	AG-27B-0005	1:2000
Flag-tag	Sigma	F1804-50ug	1:1000
IL-1α	eBioscience	14-7011-85	1:1000
SMAD2/3	Cell Signaling	#3102	1:1000
P-SMAD2	Cell Signaling	#3108	1:1000
VCAM-1	R&D Systems	AF643	1 :1000
GAPDH	Origene	TA302944	1 :10000
**Flow Cytometry Antibodies**			
CD16/32 (Fc Block)	Biolegend	101302	1 :50
CD45-PE	Biolegend	101306	1:2000
CD11b-BV605	Biolegend	101257	1:2000
Ly-6C-FITC	Biolegend	128006	1:200
F4/80-AF647	Biolegend	123122	1:200
CD206-PE/Cy7	Biolegend	141720	1:100
