# Supplementary material for: The systemic deletion of interleukin-1α reduces myocardial inflammation and attenuates ventricular remodeling in murine myocardial infarction
Source: Sci Rep. 2023 Mar 10;13:4006. doi: 10.1038/s41598-023-30662-4 (PMC10006084; doi:10.1038/s41598-023-30662-4)

Western blots images

Figure 1g

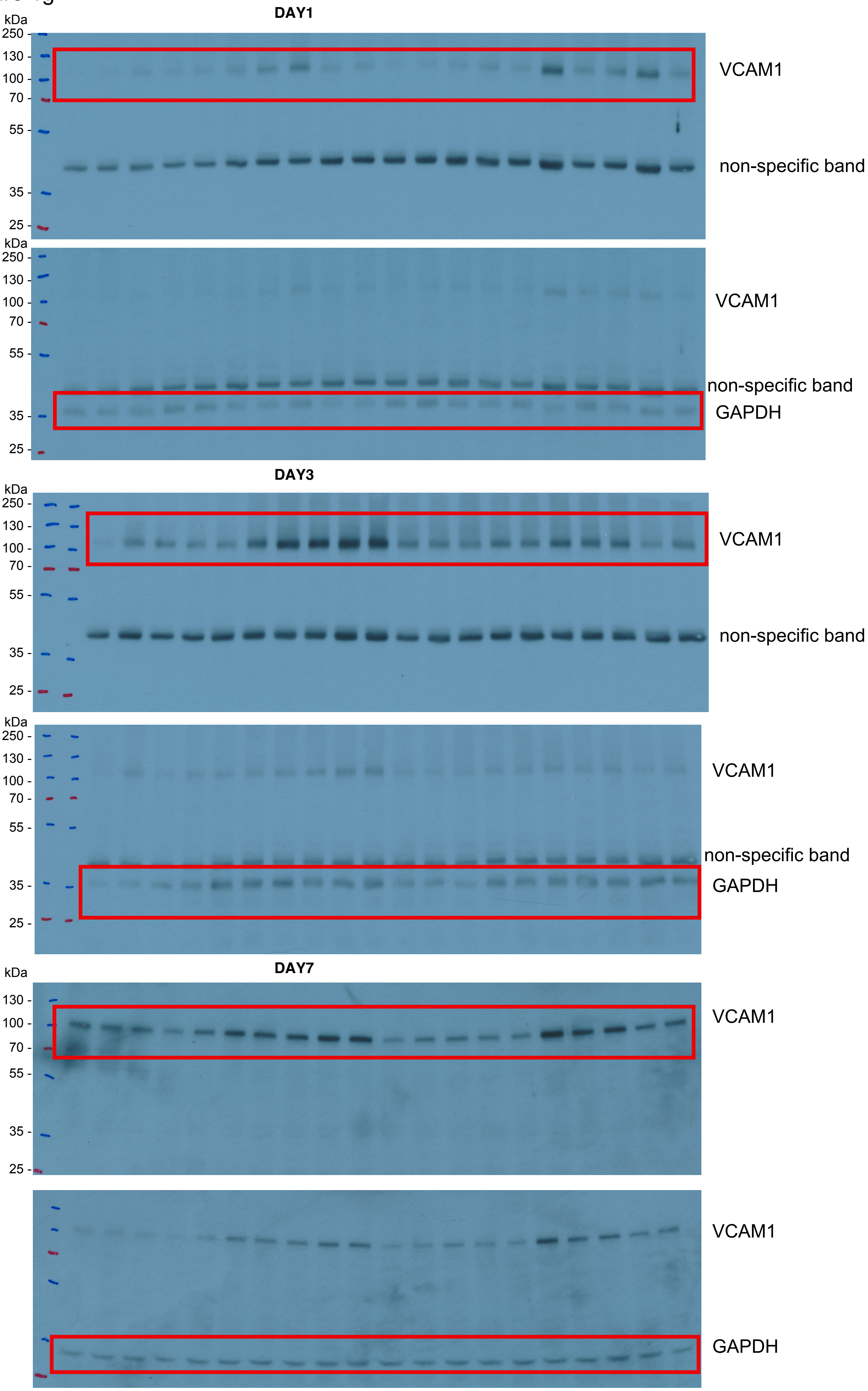

Western blots images

Figure 3a

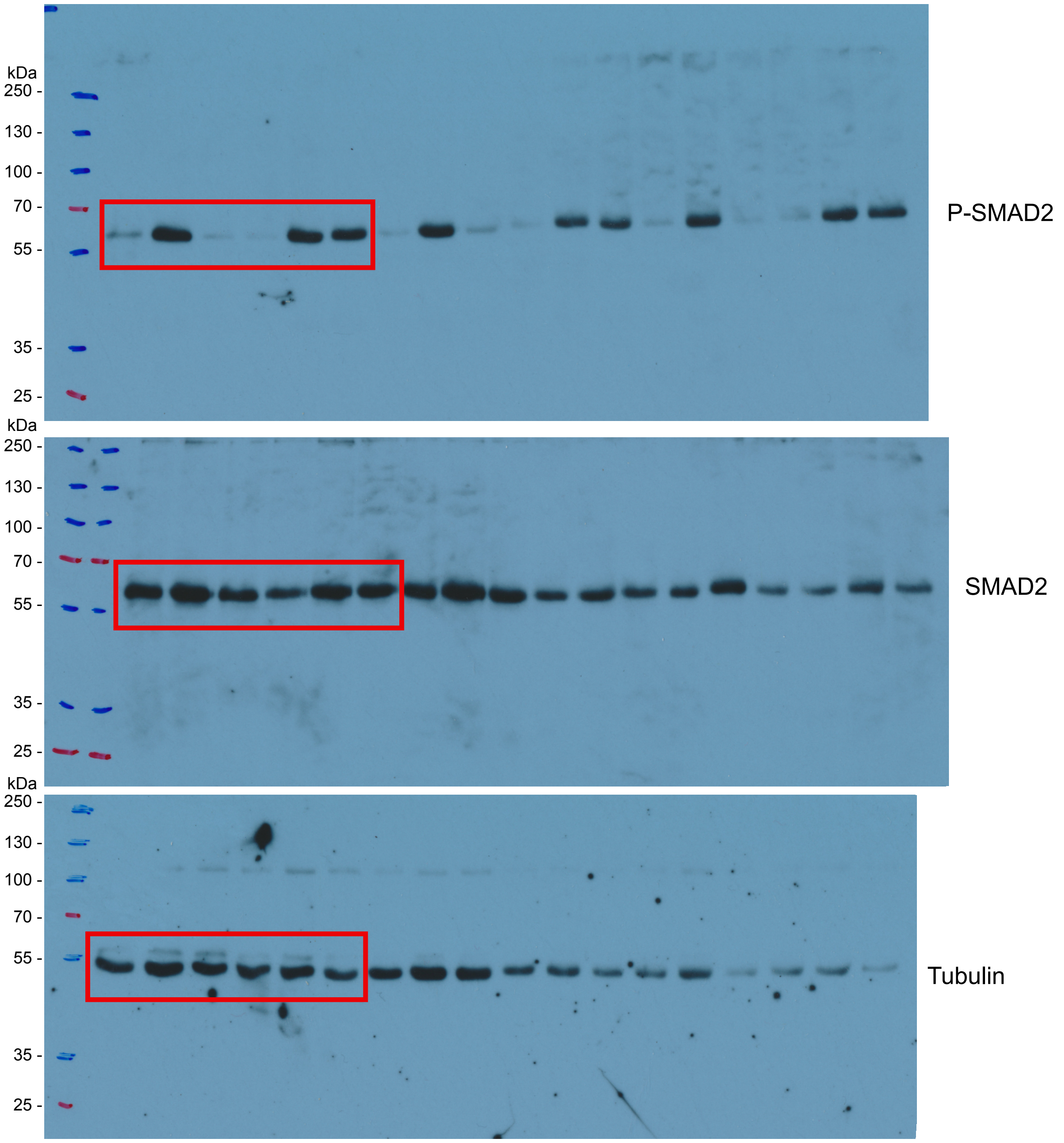

Western blots images

Figure 3b

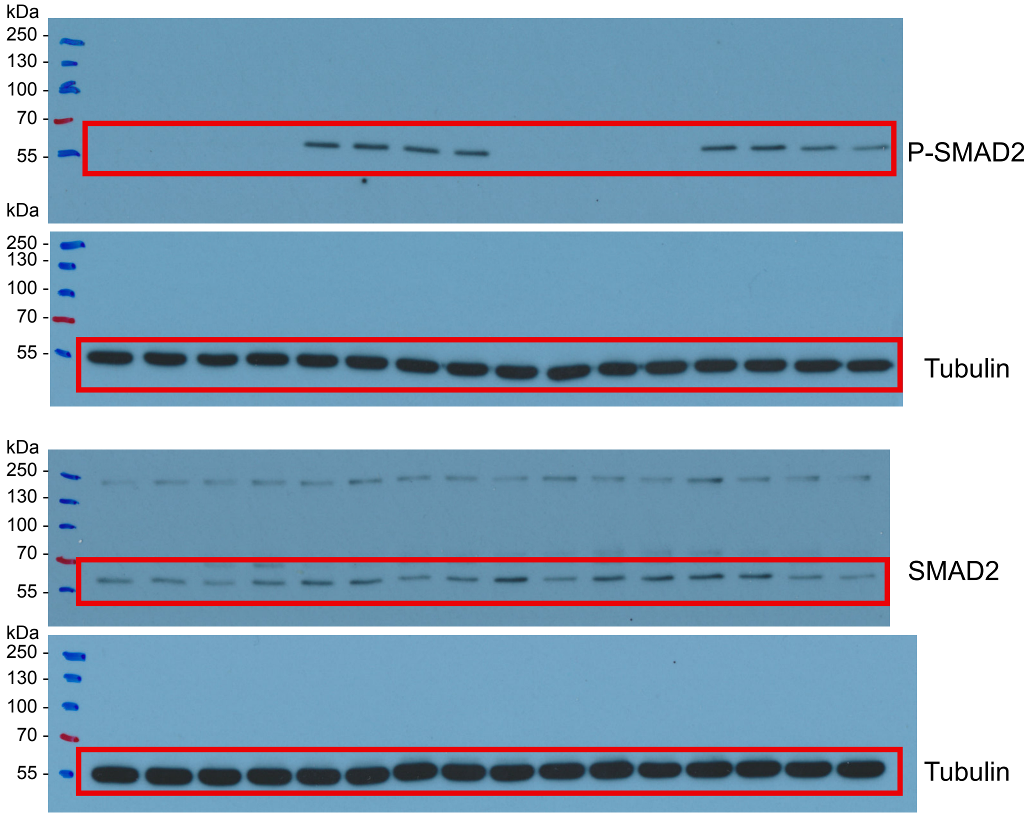

Figure 3c

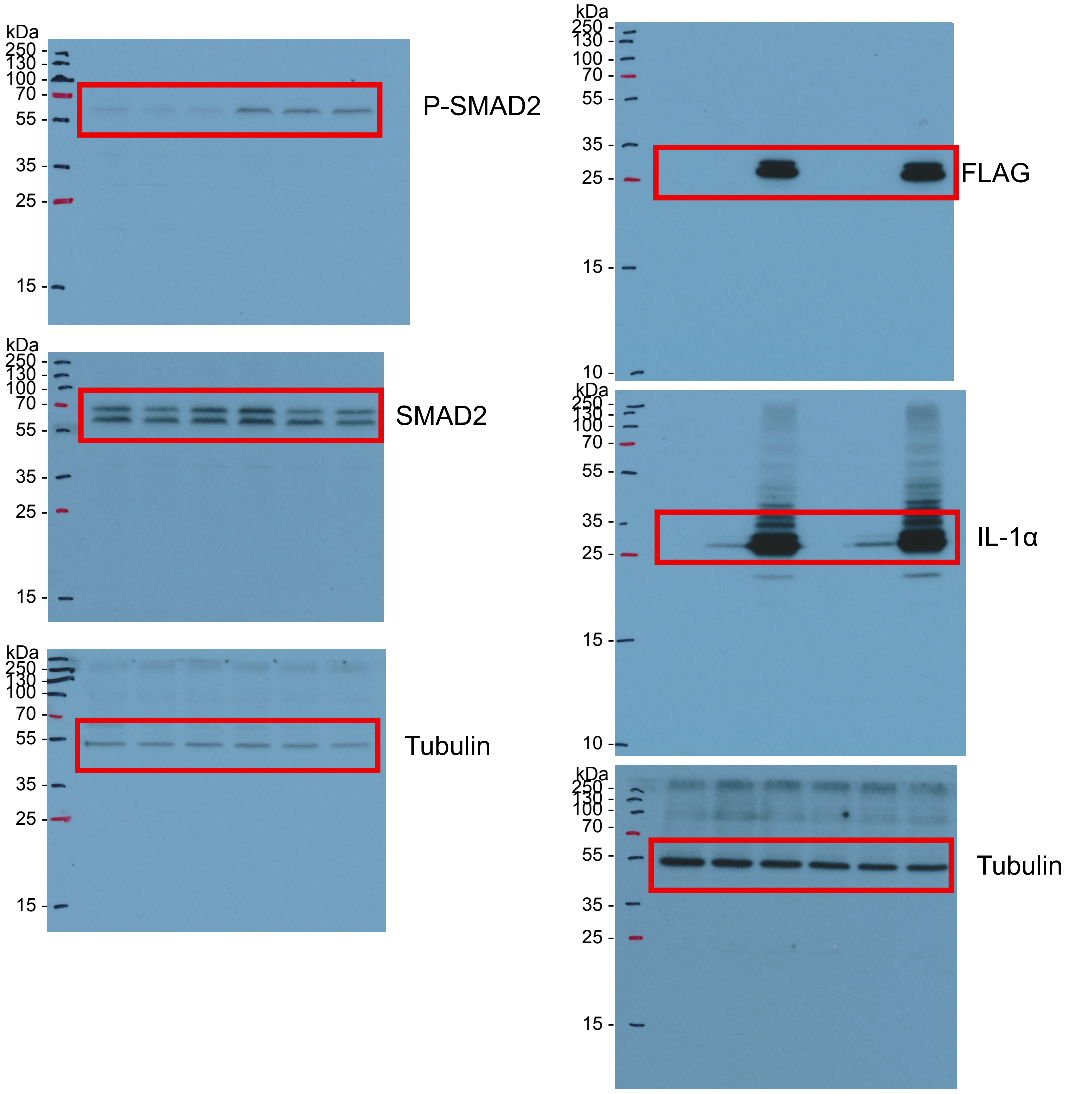

Western blots images

Figure 3h

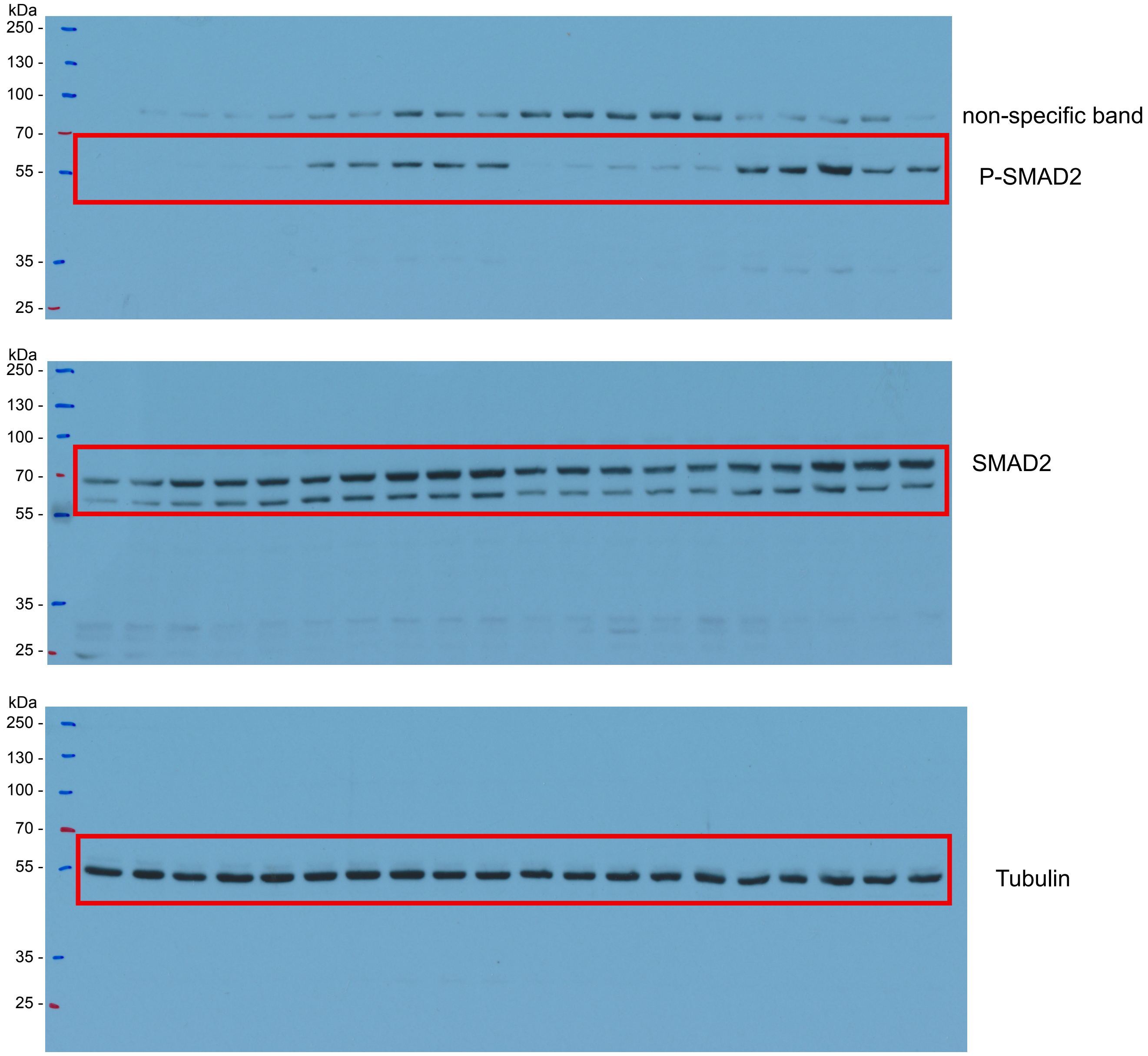

PCR images

Figure 6A

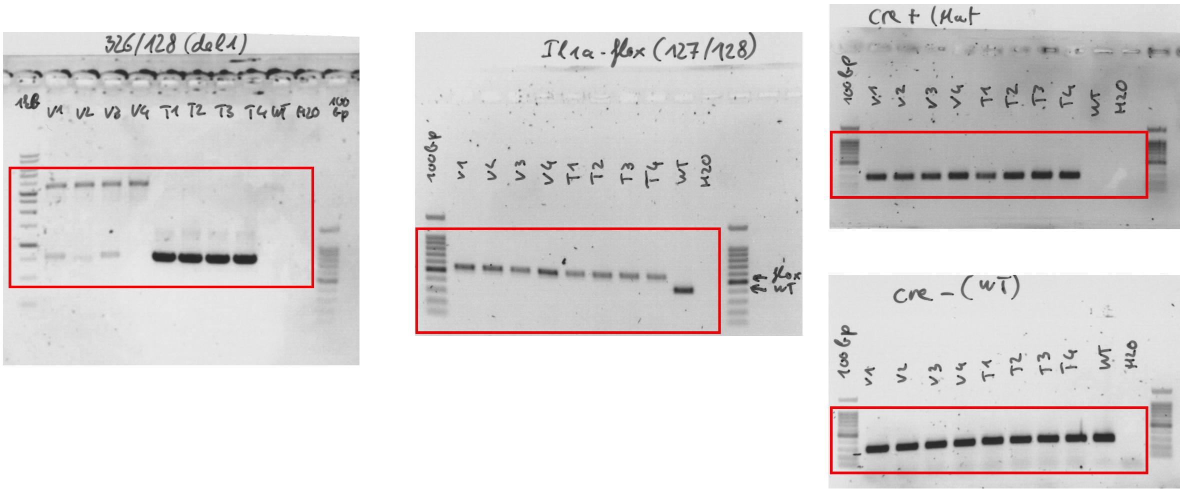

Supplement: Supplementary file 2 — Supplementary Information 2. [file 41598_2023_30662_MOESM2_ESM.pdf]
